# Causal relationship between levels of myeloperoxidase and obstructive sleep apnea: a bidirectional two-sample Mendelian randomization study

**DOI:** 10.3389/fneur.2023.1305580

**Published:** 2023-12-14

**Authors:** Weihua Tang, Fang Li, Rui Huang, Peijun Liu

**Affiliations:** ^1^Department of Respiratory and Critical Care Medicine, The Central Hospital of Enshi Tujia and Miao Autonomous Prefecture, Enshi, China; ^2^Department of Radiology, The Central Hospital of Enshi Tujia and Miao Autonomous Prefecture, Enshi, China; ^3^Department of Cardiology, The Central Hospital of Enshi Tujia and Miao Autonomous Prefecture, Enshi, China

**Keywords:** Mendelian randomization, OSA, MPO, IVW, causal relationship

## Abstract

**Background:**

Several observational studies have investigated the association between myeloperoxidase (MPO) and obstructive sleep apnea (OSA). However, the nature of this relationship remains uncertain due to potential selection and confounding biases. To resolve this, we conducted a bidirectional two-sample Mendelian randomization (MR) study to scrutinize the causal relationship between MPO and OSA.

**Methods:**

Instrumental variables (IVs) for OSA were sourced from the publicly available FinnGen dataset, encompassing 38,998 OSA cases and 336,659 controls. Data on MPO were sourced from a study of 21,758 individuals conducted by the European Bioinformatics Institute (EBI). The primary MR analysis utilized the inverse-variance weighted (IVW) method, with MR–Egger intercept and leave-one-out methods assessing pleiotropy and Cochran’s *Q* test determining heterogeneity.

**Results:**

The IVW analysis indicated a causal relationship between heightened MPO levels and an increased incidence of OSA. Individuals with elevated MPO levels manifested a higher propensity to develop OSA, exhibiting an odds ratio (OR) of 1.075 and a 95% confidence interval (CI) of 1.011–1.143 (*p* = 0.021). Conversely, the reciprocal analysis unveiled no significant association between OSA and heightened MPO levels (*p* = 0.643). No directional pleiotropy was identified through the MR–Egger intercept test (*p* > 0.05).

**Conclusion:**

Our study provides evidence of an association between elevated MPO levels and an increased incidence of OSA. However, OSA does not necessarily lead to elevated MPO levels. When patients present with high MPO levels, screening for OSA may be advisable, considering their clinical characteristics.

## Introduction

Obstructive sleep apnea (OSA) is a widespread sleep-related respiratory disorder impacting a substantial number of individuals globally, with prevalence rates varying between 9 and 38% (1). It has significant detrimental effects on both individuals and society. The primary characteristic of OSA is the recurrent obstruction of the upper airway during sleep, resulting from the relaxation of throat muscles and leading to partial or complete pauses in breathing and reduced airflow. Common symptoms include snoring, excessive daytime fatigue, morning headaches, difficulties in concentration, memory impairment, and decreased libido (2, 3). OSA is significantly linked to smoking. As a key risk factor, smoking can increase the likelihood of developing OSA by inducing inflammation and swelling in the upper respiratory tract, altering sleep architecture, affecting neural regulation, and causing increased relaxation of the pharyngeal muscles (4, 5). The diagnosis of OSA is primarily based on monitoring the apnea-hypopnea index (AHI) during a polysomnography test (6). The fundamental pathophysiological process propelling OSA is intermittent hypoxia stemming from repeated incidents of airway blockage while sleeping. This results in recurrent awakenings in the brain, sympathetic nervous system stimulation, and reduced blood oxygen saturation levels (7).

Myeloperoxidase (MPO), a constituent of the peroxidase enzyme family and released by activated white blood cells, functions as a significant marker for oxidative distress in inflammatory conditions (8).MPO has been implicated in endothelial dysfunction, atherosclerosis development, and vascular injuries (9). Research has elucidated a significant link between raised levels of MPO and an enhanced risk of ischemic stroke, with recent investigations also associating MPO with the onset of heart failure, a crucial contributing factor to the development of ischemic stroke (10). Liao et al. investigated the levels of hepatocyte growth factor (HGF) and MPO in patients with OSA and found that higher levels of both were correlated with disease severity. Surgical interventions and continuous positive airway pressure (CPAP) treatment were shown to reduce cardiovascular damage, and the combined detection of HGF and MPO provided valuable insights into disease progression and treatment efficacy (11). Ferhat et al. discovered elevated levels of MPO activity and sTNF-R1 in the plasma of OSA patients. They also found a weak but significant correlation between MPO activity and disease severity measured by the Apnea-Hypopnea Index (AHI). These discoveries imply that the augmented oxidative stress and systemic inflammation detected in patients with OSA may play a role in elevating the risk of developing cardiovascular diseases (12). However, differing opinions suggest that it might not be OSA but smoking that is substantially correlated with elevated levels of MPO and MMP-9 in patients, emphasizing the need to consider smoking status when evaluating the effects of OSA. The working mechanism might be that the intermittent hypoxia caused by OSA may only lead to cellular membrane damage without oxidative damage at the cytoplasmic and tissue levels (13). MPO levels are significantly associated with smoking, which activates neutrophils and increases MPO release. This enzyme, active in inflammation and oxidative stress, catalyzes reactive oxygen species formation, exacerbating these conditions. Therefore, smokers often have higher MPO levels (14). Therefore, the causal relationship between MPO and OSA remains a subject of ongoing debate and discussion within the scientific community.

Mendelian Randomization (MR) stands as a robust analytical tool, leveraging genetic variants as instrumental variables (IVs) to deduce causal associations between pertinent variables (15, 16). By leveraging the random assortment of genetic variations during conception, MR can help minimize biases related to confounding factors and reverse causation, commonly encountered in observational studies (17). Furthermore, according to Mendel’s second law, DNA sequences remain stable, and the transmission of biological information follows a unidirectional flow. As a result, MR can effectively minimize the potential for reverse causation and mitigate any confusion that may arise from it (18). Given the MR method’s capacity to reduce confounding bias, our study utilized a two-sample Mendelian randomization (MR) analysis to elucidate the causal relationship between MPO and OSA.

## Materials and methods

### Study design

**Figure 1:**
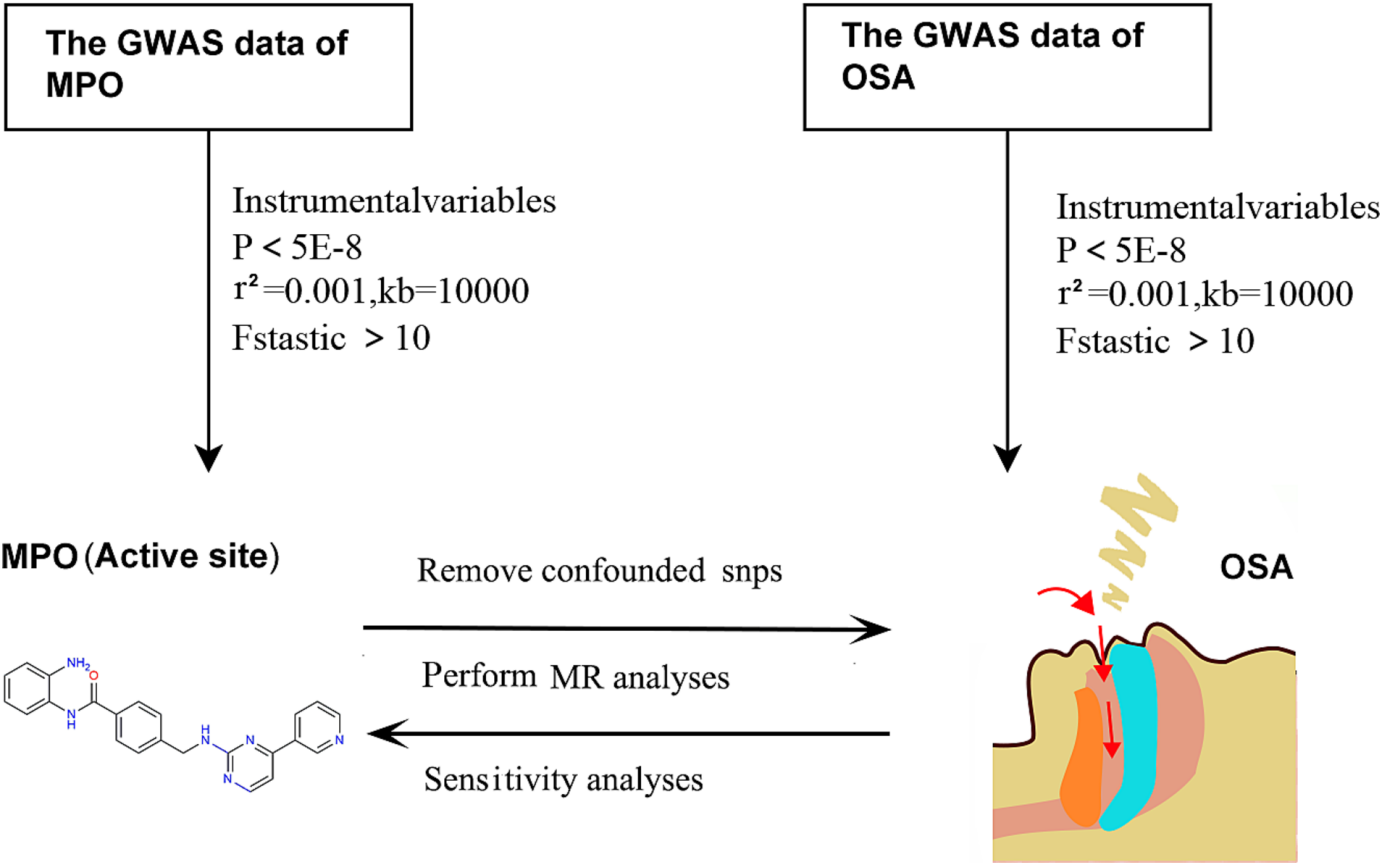
Schematic of the MR methodology used in this study. MPO, myeloperoxidase; OSA, obstructive sleep apnea; MR, Mendelian randomization.

Given that the data utilized in this study were sourced from publicly available online repositories, there was no requirement for ethical approval or informed consent. The flowchart depicting the MR methodology for this study is presented in Figure 1. To meet the prerequisites for MR analysis, the instrumental variables (IVs) selected should fulfill the following criteria: (1) The genetic markers should have a direct effect on the desired exposures. (2) There should be no association between the genetic markers and potential confounders. (3) The genetic markers should impact the outcomes solely through their influence on the exposures (19, 20). The analyses were conducted utilizing the “TwoSampleMR” package (version 0.5.6) and the “MRPRESSO” package (version 1.0) in R (version 4.2.1).To address the limitations associated with MR analysis, we exerted rigorous efforts to identify suitable genetic instruments and managed pleiotropic effects, accounted for reverse causality, and minimized the impact of confounding variables to ensure the robustness and reliability of our findings.

Genetic instruments used to measure plasma MPO levels.

The data on MPO were obtained from a study examining protein quantitative trait loci (pQTLs) associated with 90 cardiovascular proteins in a large cohort of over 30,000 individuals of European descent. This study identified a total of 451 pQTLs for 85 proteins. To validate the findings, pathway mapping, mouse knockdown studies, and clinical evaluations were undertaken. The study also evaluated known drug targets and utilized Mendelian randomization to propose new candidate targets for repositioning. Subsequently, 11 proteins revealed causal connections to human disorders that were previously unexplored. These findings highlight the significance of extensive proteome genetic mapping in precision studies focusing on circulating proteins and their impact on human health (21).

### Data sources of OSA

The IVs for OSA were derived from the FinnGen database, which can be publicly accessed at https://storage.googleapis.com/finngen-public-data-r9/. This extensive dataset consists of information from 38,998 individuals diagnosed with OSA and 336,659 control subjects. It is worth noting that the data in the FinnGen database predominantly represent individuals of European descent (22).

### Selection of IVs

The selection of appropriate IVs involves meeting several criteria. Firstly, single nucleotide polymorphisms (SNPs) should exhibit a significant association with the target exposure at a genome-wide significance threshold (*p* < 5E-08) (23). Secondly, the SNPs should be assessed for linkage disequilibrium using the PLINK algorithm to ensure a low level of correlation (*r*^2^ < 0.001) within a clumping window size of 10,000 kb (24). In the third step, the PhenoScanner GWAS database^1^ is consulted to manually exclude any IVs associated with outcome traits (25). Lastly, SNPs possessing an F statistic under 10 are deemed as weak instruments and are thus omitted from subsequent analysis (26).

### Statistical analysis

This study utilized several analytical techniques, including inverse variance weighted (IVW), MR-Egger, weighted median, and weighted mode, to examine the causal association between MPO and OSA. The IVW method was chosen as the primary analytical approach, assuming that each SNP serves as a valid instrumental variable (IV). The IVW method was preferred due to its favorable statistical performance compared to other MR methodologies (27).

## Results

The Impact of Myeloperoxidase on Obstructive Sleep Apnea.

**Figure 2:**
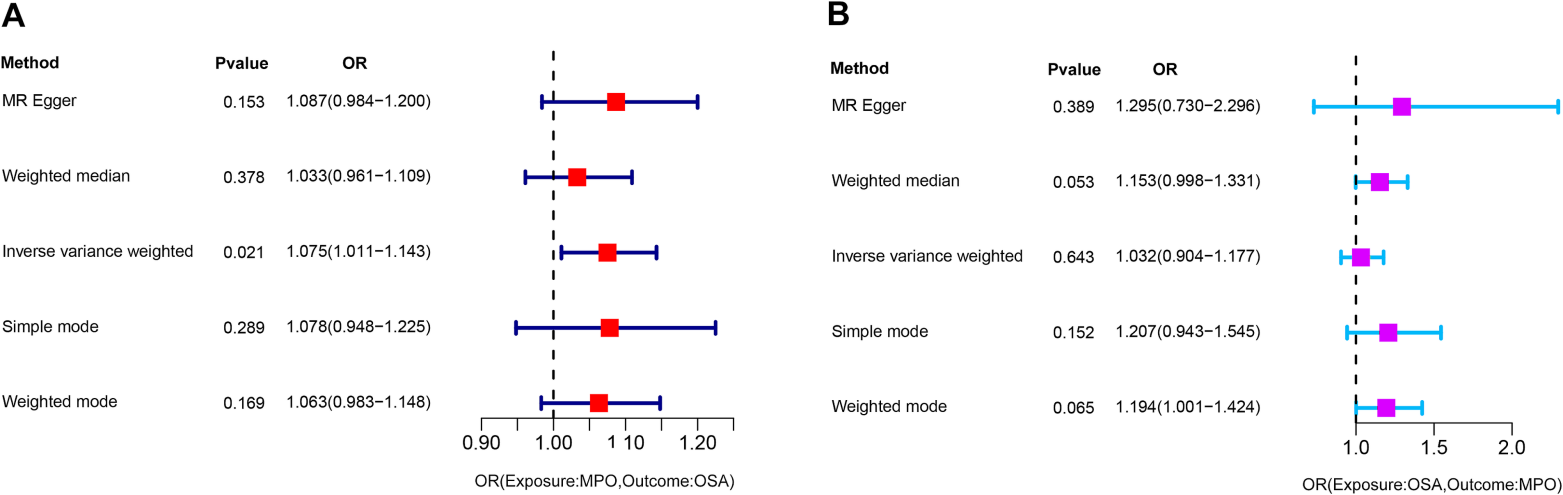
Graphical representation of the IVW analysis results for assessing the causal association. **(A)** A significant causal association between exposure to MPO and the incidence of OSA (*p* = 0.021). **(B)** Analysis shows no significant causal association between OSA and exposure to MPO (*p* = 0.643). MPO, myeloperoxidase; OSA, obstructive sleep apnea; OR: odds ratio.

In our study, we examined the impact of MPO on OSA and identified specific SNPs across the genome that demonstrated a significant association with MPO. We carefully assessed the presence of linkage disequilibrium among these SNPs. After excluding palindromic SNPs and those related to smoking and obesity, we identified 9 SNPs that served as IVs for our analysis (Supplementary Table S1). One SNP, rs757081, was excluded due to pleiotropy. The F-statistic for each IV exceeded 10, indicating strong validity for all selected instrumental variables. The primary analysis using the IVW approach suggested a significant causal association between exposure to MPO and the incidence of OSA (OR: 1.075, 95% CI: 1.011–1.143, *p* = 0.021) (Figure 2A). However, the analyses conducted using MR–Egger, weighted median, simple mode, and weighted mode did not yield considerable evidence to back a notable causal link between exposure to MPO and OSA.

**Figure 3:**
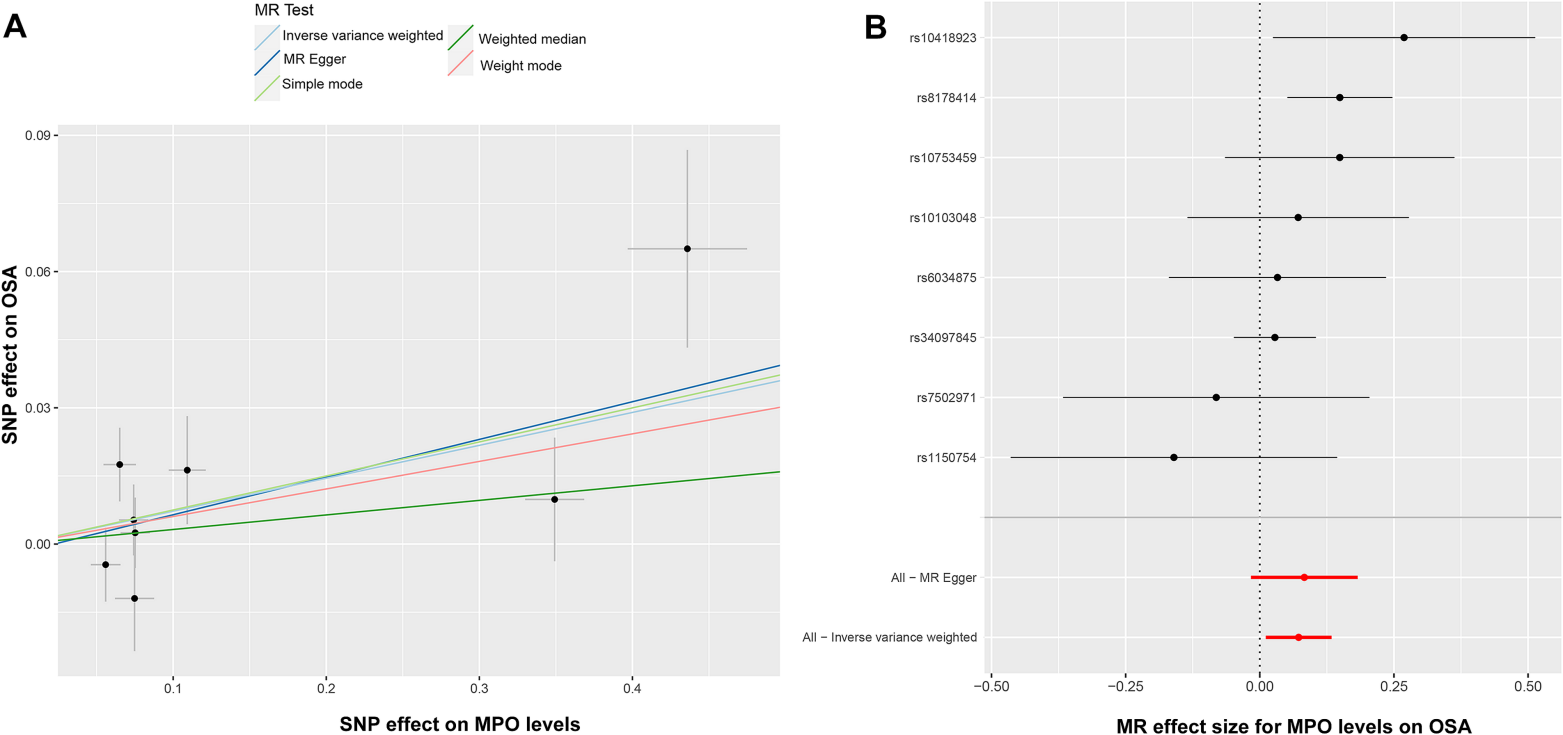
Illustration of the causal analysis between elevated MPO levels and OSA. **(A)** Scatter plots suggest a potential association between elevated MPO levels and OSA. **(B)** The forest plot, with black dots for SNP estimates and a red line for the MR-Egger result, validates the IVW method’s robustness and pleiotropy control. MPO, myeloperoxidase; OSA, obstructive sleep apnea; IVW, inverse-variance weighted.

Nevertheless, it is noteworthy that the OR values from these methods consistently indicated that elevated MPO levels might potentially contribute to the development of OSA. In the context of Mendelian randomization analysis, compared to the weighted median, weighted mode, simple mode, and MR–Egger approaches, the IVW method usually delivers more stable and trustworthy evaluations of causal connections owing to its elevated statistical potency and improved bias mitigation (28). Heterogeneity was detected using Cochran’s *Q* test (*Q* = 10.004, *p* = 0.124). The MR–Egger intercept analysis revealed no evidence of pleiotropy (*p* = 0.439). The corresponding scatter plot and forest plot illustrating these findings can be found in Figures 3A,B. The Leave-one-out analysis is a rigorous method used to evaluate the performance of a model. It systematically validates the model by iteratively excluding individual data points and assessing its accuracy using the remaining data (29). Supplementary Figure S1A provides insight into the model’s performance during each round of leave-one-out cross-validation. This plot indicates the reliability and robustness of the analysis results for the association between MPO and OSA. The funnel plot in Supplementary Figure S1B suggests that there is no evidence of bias or heterogeneity, indicating the reliability of the results and the relationship between MPO and OSA.

The Impact of Obstructive Sleep Apnea on Myeloperoxidase.

**Figure 4:**
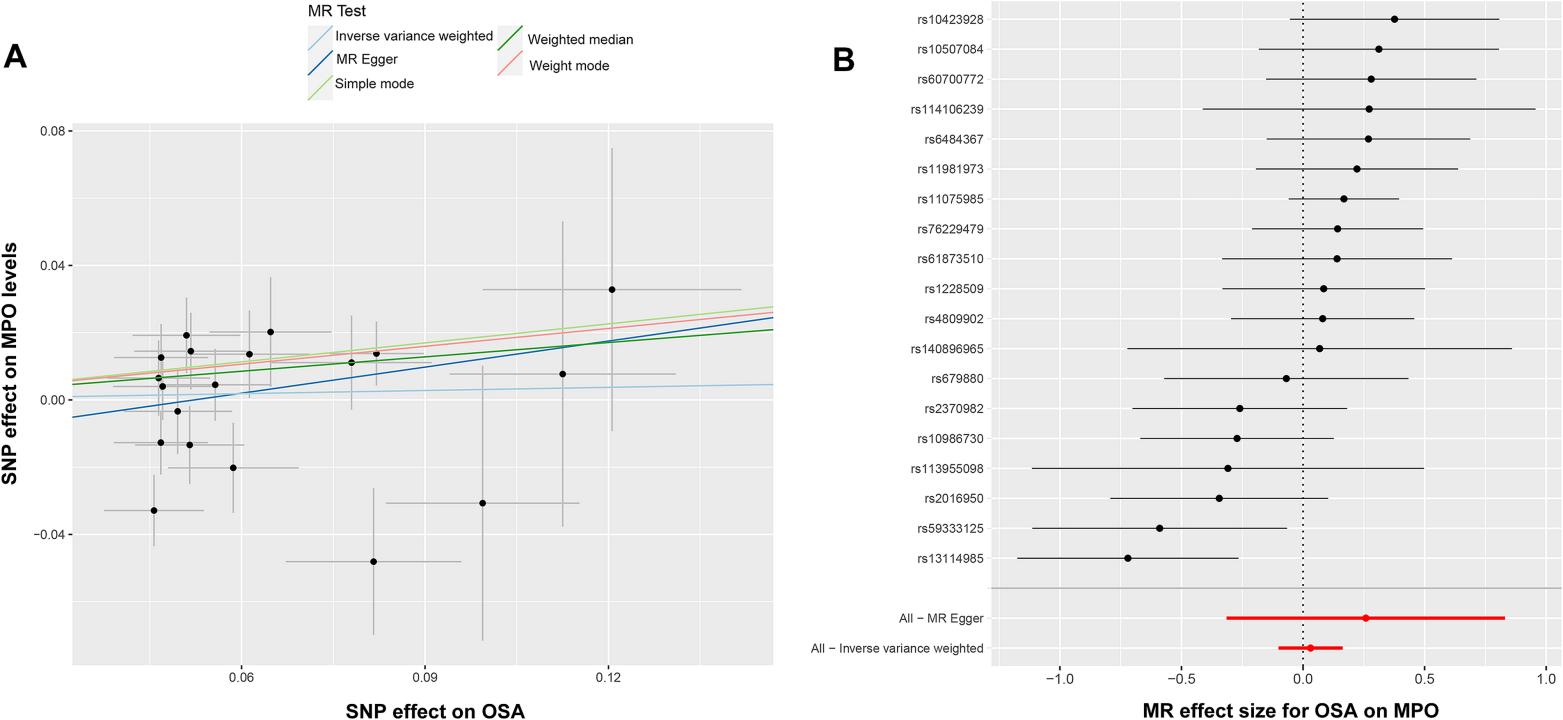
Illustration of the analysis of causal inference between OSA and MPO. **(A)** Scatter plot indicating no discernible association between elevated OSA and MPO levels. **(B)** The forest plot emphasizes the reliability of the IVW method, with black dots representing individual SNP, and a red line indicating the aggregate MR-Egger estimate. MPO, myeloperoxidase; OSA, obstructive sleep apnea; and IVW, inverse-variance weighted.

To investigate the potential impact of OSA on MPO, a reverse Mendelian randomization analysis was conducted. SNPs with strong associations to OSA were chosen, and post adjustment for potential confounders like smoking and obesity, 20 SNPs were discerned as instrumental variables for the examination (Supplementary Table S2). However, due to the presence of pleiotropy, rs13333522 was excluded from the set of IVs. The IVW analysis did not indicate a significant causal association between OSA and MPO exposure (OR: 1.032, 95% CI: 0.904–1.177, *p* = 0.643) (Figure 2B). The MR–Egger results showed a more significant odds ratio (OR: 1.295, 95% CI: 0.730–2.296, *p* = 0.389), suggesting a potential directional association. Similarly, the weighted mode and weighted median analyses presented odds ratios above 1, indicating a possible positive association between OSA and MPO levels. Significant heterogeneity was detected based on Cochran’s *Q* test (*Q* = 31.767, *p* = 0.161), while the MR–Egger intercept analysis indicated no evidence of pleiotropy (*p* = 0.435). These findings are illustrated in the corresponding scatter and forest plots (Figures 4A,B). The leave-one-out plot demonstrated a positive correlation, suggesting that there is a potential association between increased levels of OSA and elevated MPO levels (Supplementary Figure S2A). Additionally, the funnel plot did not indicate any significant bias or heterogeneity in the relationship between OSA and MPO (Supplementary Figure S2B).

## Discussion

To our understanding, this study is the first to employ Mendelian randomization (MR) analysis in investigating the causal connection between MPO levels and OSA in individuals of European descent. The findings indicate that increased MPO concentrations are correlated with a raised likelihood of developing OSA. However, it’s essential to clarify that experiencing OSA does not definitively correspond to elevated levels of MPO. These revelations highlight the potential involvement of MPO in the evolution of OSA and accentuate the necessity for continued exploration in this field.

There are several inflammatory markers associated with OSA, including C-reactive protein (CRP). A meta-analysis of five studies, involving 219 OSA participants and 116 controls, found that the OSA group had significantly higher levels of high-sensitivity CRP compared to the control group. CRP is known to be associated with systemic inflammatory responses. The objective of our study is to identify a biomarker that correlates with both cardiac diseases and OSA (30). Meltem et al. examined the clinical relevance of MPO in both serum and saliva, along with serum levels of CRP, as indicators of inflammation in OSA. The research showed a significant elevation in salivary MPO and serum CRP levels in subjects with OSA in comparison to those in the control group. The findings support the idea of persistent local inflammation in OSA patients, highlighting the potential value of salivary MPO levels as indicators of oropharyngeal inflammation in OSA (31).In clinical scenarios, OSA is closely associated with cardiovascular diseases, and Continuous CPAP therapy is a primary treatment approach. In a study involving 153 participants with OSA, 76 individuals were randomly assigned to receive CPAP treatment, while the remaining 77 were assigned to a control group receiving sham CPAP. Both groups underwent the respective interventions for a duration of 2 months. Following the treatment period, the results revealed no significant changes in oxidative stress markers, including MPO and F2-Isoprostanes (32). Activated polymorphonuclear leukocytes play a crucial role in developing and progressing smoking-induced vascular inflammation and atherosclerosis. This sequence triggers the liberation of MPO, influencing the decrease in bioavailability of endothelial nitric oxide, inciting the oxidation of both low and high-density lipoproteins (LDL and HDL), and advancing the development and instability of atherosclerotic plaques (33, 34).

The potential association between smoking and OSA remains uncertain, presenting conflicting findings across various studies. Smoking correlates with insomnia, manifesting in increased awakenings, extended N1 and N2 sleep stages durations, and a decrease in deep sleep phases. Individuals suffering from undiagnosed or untreated OSA may resort to smoking to leverage the stimulating effects of nicotine, especially those exhibiting symptoms of depression (35). The definitive connection between MPO and OSA is yet to be elucidated due to the interference of confounding elements like smoking and BMI, and the diverse severity levels of AHI. These elements add layers of complexity in deciphering the true connection between MPO and OSA. MR analysis is adept at sidestepping the inherent confounders and biases of reverse causation present in observational studies (36). Additionally, it can overcome the high cost and limited practicability of randomized controlled trials. Consequently, MR analysis is a potent tool capable of furnishing robust evidence illustrating a causal association between MPO and OSA.

MPO, a peroxidase enzyme laden with heme, is chiefly located in neutrophils and to a lesser extent in monocytes. It represents a pivotal enzyme expelled from the secondary granules following the activation of neutrophils (37, 38). While the oxidants generated by MPO are beneficial in immune responses against invading pathogens, substantial evidence suggests that inappropriate oxidant generation stimulation may lead to host tissue damage (39).In individuals with OSA, heightened levels of oxidative stress and diminished antioxidant activity have been observed compared to those without OSA (40). Ahmet et al. included a total of 70 participants in their study: 50 with OSA (comprising 11 mild cases making up 6.9%, 17 moderate cases accounting for 24.7%, and 22 severe cases representing 68.5%) and 20 individuals exhibiting simple snoring as the control group. The outcomes revealed no significant disparities in MPO across all groups (41). This indicates that the AHI index we use for categorizing OSA does not maintain a close correlation with MPO either. The mentioned studies align with our research findings, implying a potential association between MPO and OSA. However, it is essential to note that not all individuals with OSA will necessarily have elevated levels of MPO.

In our MR study, the genetic data for exposure and outcome were sourced from populations of European descent. This approach helps to minimize biases arising from racial differences and reduces the potential for sample overlap. The GWAS datasets used in our analysis involved large case–control samples, including 30,931 participants for MPO and 375,657 participants for OSA. With many participants, our MR analysis is well-powered to detect genetic effects on outcome risks. To ensure robust results, we employed a stringent SNP selection threshold of *p* < 5E-08. This approach facilitated the identification of SNPs with more potent genetic effects to be used as IVs. Moreover, setting such a threshold helps to mitigate errors associated with multiple comparisons, reducing the likelihood of false-positive results (42). The F-statistic above ten and *p* < 0.05 for the MR–Egger intercept suggest no evidence of pleiotropy caused by IVs. In contrast, a *p* > 0.05 for the Cochran *Q* test indicates the absence of heterogeneity. However, it is crucial to recognize some constraints in this study. Initially, the insights are drawn from data focused on individuals of European origin, necessitating more extensive exploration to ascertain if the outcomes are applicable to diverse ethnic communities. Furthermore, given the gradations of OSA (mild, moderate, and severe) based on its intensity, more investigations are imperative to understand whether the recurrent intermittent hypoxia characteristic of severe OSA can escalate MPO levels.

The relationship between MPO and OSA remains not fully understood. Our MR analysis suggests a significant role of MPO in OSA development. Monitoring elevated MPO levels during check-ups or hospitalizations can aid in identifying individuals at OSA risk, as confirmed through sleep studies. For severe OSA patients, assessing and reducing elevated MPO levels is crucial. However, it’s important to recognize the limitations of this study, including potential confounding variables and the challenges posed by using tertiary databases, which may introduce biases or errors. Our exploration of the association between OSA and MPO levels lies in its potential challenge to adequately address reverse causality and unmeasured confounders. This reality underscores the importance of interpreting our findings with caution and highlights the necessity of using varied methodologies in future research to enhance our understanding of the potential causes and progression of OSA.

## Conclusion

Our bidirectional two-sample MR study elucidates the causal relationship between MPO levels and the incidence of OSA. By utilizing instrumental variables derived from large datasets, our findings provide robust evidence for an increased risk of OSA associated with elevated MPO levels. The odds ratio indicates that individuals with higher MPO levels are more likely to develop OSA. However, we did not find evidence supporting the reciprocal association, suggesting that OSA does not inherently lead to elevated MPO levels.

## Data availability statement

The original contributions presented in the study are included in the article/Supplementary material, further inquiries can be directed to the corresponding authors.

## Ethics statement

Ethical review and approval was not required for the study on human participants in accordance with the local legislation and institutional requirements. Written informed consent from the patients/participants or patients/participants’ legal guardian/next of kin was not required to participate in this study in accordance with the national legislation and the institutional requirements.

## Author contributions

WT: Data curation, Investigation, Software, Writing – original draft. FL: Methodology, Software, Writing – original draft. RH: Funding acquisition, Project administration, Writing – review & editing. PL: Conceptualization, Formal analysis, Methodology, Validation, Writing – review & editing.

## Footnotes

^1^http://phenoscanner.medschl.cam.ac.uk

## Supplementary material

### SUPPLEMENTARY FIGURE S1

Depiction of analytical robustness and reliability. **(A)** The plot shows the model’s performance during leave-one-out cross-validation, highlighting the reliability and robustness of the analysis. **(B)** The funnel plot shows no discernible bias or heterogeneity, affirming the reliable association between MPO and OSA. MPO, myeloperoxidase; OSA, obstructive sleep apnea.

### SUPPLEMENTARY FIGURE S2

Illustration of positive correlation and analytical consistency. **(A)** The leave-one-out plot indicates a positive correlation, showcasing a potential association between elevated levels of OSA and increased MPO levels. **(B)** The funnel plot revealed no significant bias or heterogeneity, underscoring the consistency in the relationship between OSA and MPO.OSA, obstructive sleep apnea, MPO, myeloperoxidase.
