# Supplementary material for: Causal relationship between levels of myeloperoxidase and obstructive sleep apnea: a bidirectional two-sample Mendelian randomization study
Source: Front Neurol. 2023 Dec 14;14:1305580. doi: 10.3389/fneur.2023.1305580 (PMC10753018; doi:10.3389/fneur.2023.1305580)
Supplement: Supplementary file 1 [file Table_1.DOCX]

| exposure | SNP | chr.outcome | effect_allele.  exposure | other_allele.  exposure | beta.  exposure | eaf.  exposure | se.exposure | pval.exposure | FSTAT |
| --- | --- | --- | --- | --- | --- | --- | --- | --- | --- |
| Myeloperoxidase levels | rs10103048 | 8 | C | A | -0.0742 | 0.5849 | 0.0096 | 1.14E-14 | 59.73452605 |
| Myeloperoxidase levels | rs10418923 | 19 | G | A | 0.0651 | 0.7081 | 0.0106 | 7.50E-10 | 37.71467107 |
| Myeloperoxidase levels | rs10753459 | 1 | A | G | 0.1092 | 0.1753 | 0.0121 | 1.43E-19 | 81.43940909 |
| Myeloperoxidase levels | rs1150754 | 6 | T | C | -0.0748 | 0.152 | 0.0128 | 5.87E-09 | 34.14627504 |
| Myeloperoxidase levels | rs34097845 | 17 | T | C | -0.3492 | 0.0566 | 0.0193 | 2.70E-73 | 327.3361196 |
| Myeloperoxidase levels | rs6034875 | 20 | A | G | -0.0752 | 0.5824 | 0.0096 | 4.00E-15 | 61.35547078 |
| Myeloperoxidase levels | rs7502971 | 17 | A | C | -0.0559 | 0.3703 | 0.0097 | 9.22E-09 | 33.20780919 |
| Myeloperoxidase levels | rs757081 | 11 | G | C | 0.0982 | 0.3355 | 0.0099 | 4.26E-23 | 98.38132427 |
| Myeloperoxidase levels | rs8178414 | 17 | T | C | -0.4359 | 0.0218 | 0.039 | 4.86E-29 | 124.9121265 |

TableS1| Details of the SNPs used for the MR analysis assessing the impact of MPO on OSA.
