# Supplementary material for: Causal relationship between levels of myeloperoxidase and obstructive sleep apnea: a bidirectional two-sample Mendelian randomization study
Source: Front Neurol. 2023 Dec 14;14:1305580. doi: 10.3389/fneur.2023.1305580 (PMC10753018; doi:10.3389/fneur.2023.1305580)
Supplement: Supplementary file 2 [file Table_2.DOCX]

TableS2| Details of the SNPs used for the MR analysis assessing the impact of OSA on MPO.

| Exposure | SNP | chr | effect_allele.  exposure | other_allele.  exposure | beta.  exposure | eaf.  exposure | se.exposure | pval.exposure | F |
| --- | --- | --- | --- | --- | --- | --- | --- | --- | --- |
| OSA | rs10423928 | 19 | A | T | -0.050987 | 0.258578 | 0.00882387 | 7.55E-09 | 374.8247392 |
| OSA | rs10507084 | 12 | T | C | 0.0647465 | 0.179407 | 0.00996658 | 8.23E-11 | 464.2536713 |
| OSA | rs10986730 | 9 | T | C | -0.0467971 | 0.524197 | 0.00769035 | 1.16E-09 | 410.82177 |
| OSA | rs11075985 | 16 | A | C | 0.0820534 | 0.428739 | 0.00771867 | 2.15E-26 | 1243.009847 |
| OSA | rs113955098 | 10 | A | G | -0.099427 | 0.0674969 | 0.0158313 | 3.38E-10 | 468.0613065 |
| OSA | rs114106239 | 3 | T | C | -0.120592 | 0.0371118 | 0.0211507 | 1.19E-08 | 390.8369903 |
| OSA | rs11981973 | 7 | G | A | 0.061286 | 0.181587 | 0.00984022 | 4.72E-10 | 419.8402791 |
| OSA | rs1228509 | 1 | C | A | 0.0470936 | 0.656248 | 0.00811164 | 6.41E-09 | 376.262517 |
| OSA | rs13114985 | 4 | G | T | 0.0456779 | 0.33828 | 0.00814065 | 2.01E-08 | 351.2269843 |
| OSA | rs13333522 | 16 | G | C | 0.0423543 | 0.526626 | 0.00771747 | 4.06E-08 | 336.2865444 |
| OSA | rs140896965 | 20 | T | C | -0.112519 | 0.0490272 | 0.0184655 | 1.11E-09 | 444.0062621 |
| OSA | rs2016950 | 12 | T | C | -0.0586327 | 0.157983 | 0.0106896 | 4.13E-08 | 343.8963525 |
| OSA | rs2370982 | 14 | T | C | 0.0515131 | 0.238198 | 0.00892902 | 7.97E-09 | 362.1205791 |
| OSA | rs4809902 | 20 | C | G | -0.0556845 | 0.228498 | 0.00921496 | 1.51E-09 | 411.1329914 |
| OSA | rs59333125 | 12 | C | A | -0.0815898 | 0.0807381 | 0.0143562 | 1.32E-08 | 371.5677811 |
| OSA | rs60700772 | 6 | C | T | 0.0516943 | 0.221225 | 0.00923826 | 2.20E-08 | 346.2188994 |
| OSA | rs61873510 | 10 | T | G | 0.0464319 | 0.301035 | 0.0084028 | 3.28E-08 | 341.1290476 |
| OSA | rs6484367 | 11 | A | G | 0.0468437 | 0.502695 | 0.00769204 | 1.13E-09 | 412.5966368 |
| OSA | rs679880 | 9 | A | G | 0.0495476 | 0.745023 | 0.00889332 | 2.53E-08 | 350.7037111 |
| OSA | rs76229479 | 2 | C | A | -0.0779901 | 0.0987789 | 0.0131014 | 2.64E-09 | 407.252942 |
