# Supplementary figures and images for: Causal relationship between levels of myeloperoxidase and obstructive sleep apnea: a bidirectional two-sample Mendelian randomization study
Source: Front Neurol. 2023 Dec 14;14:1305580. doi: 10.3389/fneur.2023.1305580 (PMC10753018; doi:10.3389/fneur.2023.1305580)

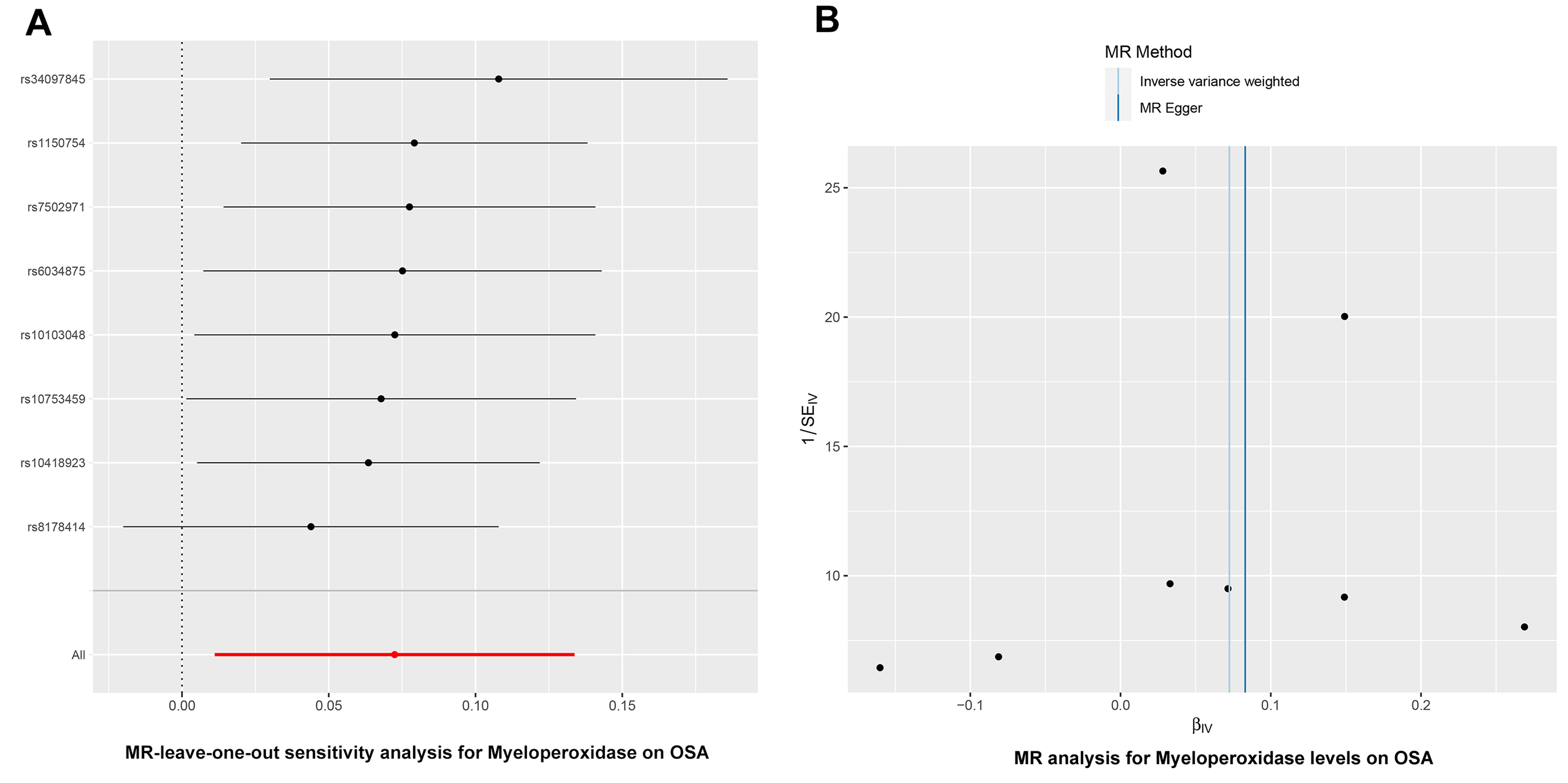

Supplement: Supplementary file 3 [file Image_1.TIF]

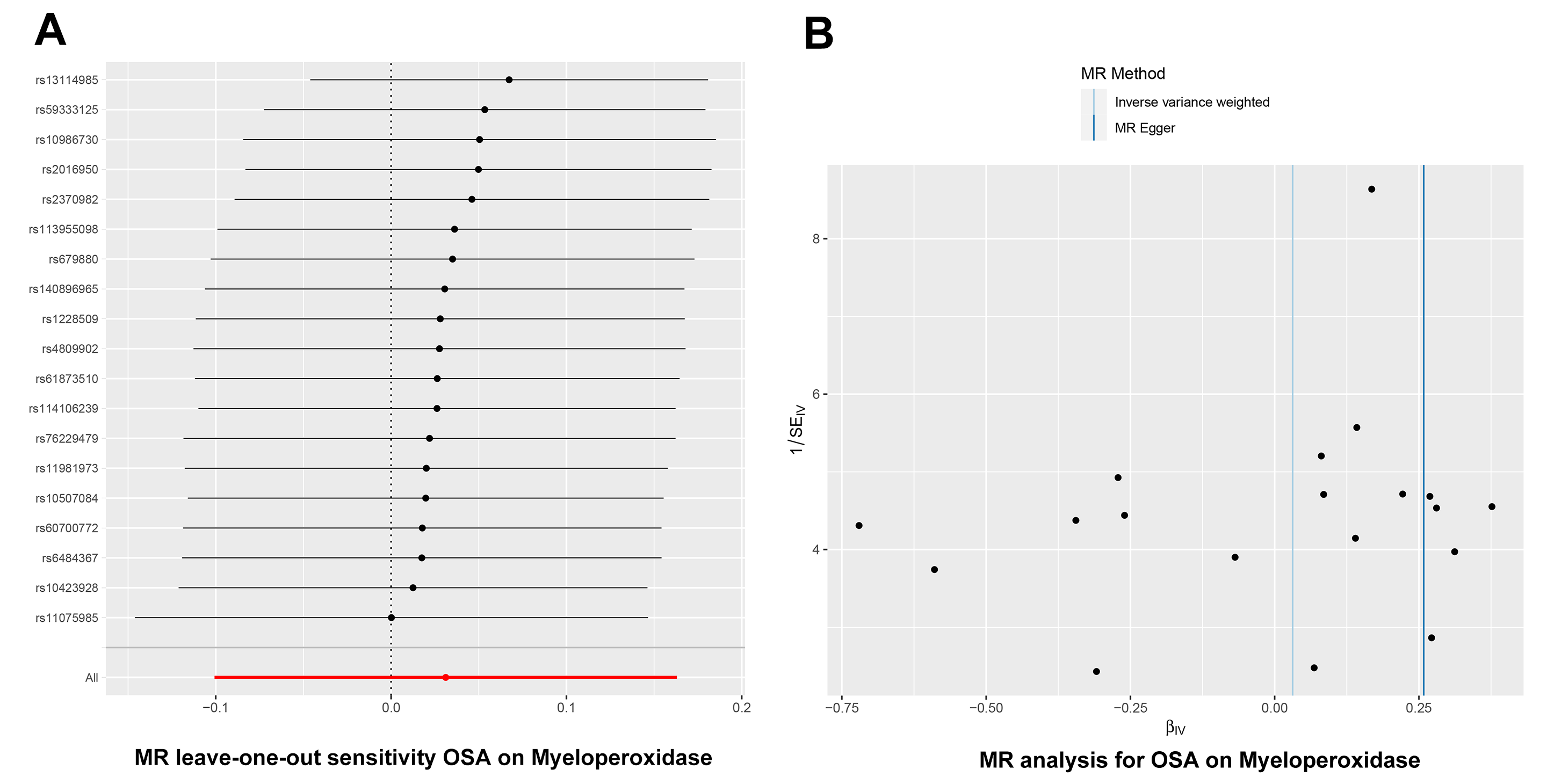

Supplement: Supplementary file 4 [file Image_2.TIF]
